# β-Elemene Reverses Gefitinib Resistance in NSCLC Cells by Inhibiting lncRNA H19-Mediated Autophagy

**DOI:** 10.3390/ph17050626

**Published:** 2024-05-14

**Authors:** Ruonan Zhang, Yintao Zheng, Qianru Zhu, Xiaoqing Gu, Bo Xiang, Xidong Gu, Tian Xie, Xinbing Sui

**Affiliations:** 1 Key Laboratory of Carcinogenesis and Cancer Invasion of the Chinese Ministry of Education, Cancer Research Institute and School of Basic Medical Sciences, Central South University, Changsha 410008, China; 20223062@hznu.edu.cn (R.Z.); xiangbolin@csu.edu.cn (B.X.); 2 School of Pharmacy, Hangzhou Normal University, Hangzhou 311121, China; zyt@hznu.edu.cn (Y.Z.); 21098533cw30001@student.must.edu.mo (Q.Z.); 2023111027024@stu.hznu.edu.cn (X.G.); xbs@hznu.edu.cn (T.X.); 3 Key Laboratory of Elemene Class Anti-Cancer Chinese Medicines, Engineering Laboratory of Development and Application of Traditional Chinese Medicines, Collaborative Innovation Center of Traditional Chinese Medicines of Zhejiang Province, Hangzhou Normal University, Hangzhou 311121, China; 4 School of Pharmaceutical Sciences, Zhejiang Chinese Medical University, Hangzhou 311402, China; 5 Department of Breast Surgery, The First Affiliated Hospital of Zhejiang Chinese Medical University, Hangzhou 310002, China

**Keywords:** β-elemene, gefitinib, autophagy, lncRNA H19, non-small cell lung cancer

## Abstract

Lung cancer is a leading cause of mortality worldwide, especially among Asian patients with non-small cell lung cancer (NSCLC) who have epidermal growth factor receptor (EGFR) mutations. Initially, first-generation EGFR tyrosine kinase inhibitors (TKIs) are commonly administered as the primary treatment option; however, encountering resistance to these medications poses a significant obstacle. Hence, it has become crucial to address initial resistance and ensure continued effectiveness. Recent research has focused on the role of long noncoding RNAs (lncRNAs) in tumor drug resistance, especially lncRNA H19. β-elemene, derived from *Curcuma aromatic* Salisb., has shown strong anti-tumor effects. However, the relationship between β-elemene, lncRNA H19, and gefitinib resistance in NSCLC is unclear. This study aims to investigate whether β-elemene can enhance the sensitivity of gefitinib-resistant NSCLC cells to gefitinib and to elucidate its mechanism of action. The impact of gefitinib and β-elemene on cell viability was evaluated using the cell counting kit-8 (CCK8) assay. Furthermore, western blotting and qRT-PCR analysis were employed to determine the expression levels of autophagy-related proteins and genes, respectively. The influence on cellular proliferation was gauged through a colony-formation assay, and apoptosis induction was quantified via flow cytometry. Additionally, the tumorigenic potential in vivo was assessed using a xenograft model in nude mice. The expression levels of LC3B, EGFR, and Rab7 proteins were examined through immunofluorescence. Our findings elucidate that the resistance to gefitinib is intricately linked with the dysregulation of autophagy and the overexpression of lncRNA H19. The synergistic administration of β-elemene and gefitinib markedly attenuated the proliferative capacity of resistant cells, expedited apoptotic processes, and inhibited the in vivo proliferation of lung cancer. Notably, β-elemene profoundly diminished the expression of lncRNA H19 and curtailed autophagic activity in resistant cells, thereby bolstering their responsiveness to gefitinib. Moreover, β-elemene disrupted the Rab7-facilitated degradation pathway of EGFR, facilitating its repositioning to the plasma membrane. β-elemene emerges as a promising auxiliary therapeutic for circumventing gefitinib resistance in NSCLC, potentially through the regulation of lncRNA H19-mediated autophagy. The participation of Rab7 in this dynamic unveils novel insights into the resistance mechanisms operative in lung cancer, paving the way for future therapeutic innovations.

## 1. Introduction

Lung carcinoma remains a primary cause of death related to cancer on a global scale [1]. Among the various types of lung cancer, non-small cell lung cancer (NSCLC) is highly prevalent, with a notable predominance of the subtype known as lung adenocarcinoma. It has been observed that about half of all cases in Asia have active gene alterations in the region responsible for encoding epidermal growth factor receptor (EGFR) tyrosine kinase activity [2]. Exon 19 deletion and exon 21 L858R point mutation are the most commonly observed EGFR mutations. Patients who test positive for EGFR mutations have demonstrated promising treatment responses to EGFR tyrosine kinase inhibitors (TKIs). Studies consistently demonstrate that these inhibitors outperform traditional platinum-based chemotherapy regimens by prolonging progression-free survival among affected patients [3,4]. However, a considerable proportion of individuals harboring these genetic alterations do not exhibit favorable responses to EGFR-TKI treatments, and the development of resistance typically occurs within a year for the majority of cases [5]. Both primary and acquired resistances limit the prolonged effectiveness of EGFR-TKIs, with the EGFR T790M mutation being a well-documented cause of acquired resistance, whereas the mechanisms of primary resistance remain less understood. This underscores the critical need for new anticancer agents that can counteract the resistance to EGFR-TKIs in lung cancer cells.

Autophagy, a cellular process controlled by autophagy-related genes in eukaryotes, entails the breakdown of cytoplasmic constituents and impaired organelles through lysosomal degradation [6]. It includes both basal autophagy under normal conditions and stress-induced autophagy. Basal autophagy acts as a cell-preservation mechanism, aiding in growth, development, and resistance to metabolic and oxidative stress, thereby maintaining cellular balance and product turnover. However, autophagy at high levels can lead to cell death by degrading essential cellular components [7]. Gefitinib has been shown to trigger protective autophagy, contributing to NSCLC cells’ intrinsic and acquired resistance to the drug [8]. Remarkably, curcumin has been found to effectively overcome the primary resistance to gefitinib in NSCLC cells by inducing autophagic cell death [9]. Moreover, the reversal of gefitinib resistance in NSCLC cells has been demonstrated through β-elemene-induced autophagy mediated by METTL3, an m^6^A methyltransferase [10]. Hence, the identification of autophagy-enhancing compounds in traditional Chinese medicine may offer a viable strategy to combat gefitinib resistance.

Elemenes, derived from the traditional Chinese medicinal plant *Curcuma aromatic* Salisb., are known for their potential therapeutic properties, including antiproliferative and anticancer effects [11,12]. Elemene injections have been reported to enhance the clinical outcomes of chemotherapy in NSCLC patients [13]. β-elemene, one of the primary isomers, has been extensively studied for its antitumor activities, influencing various pathways such as mitochondrial activation, apoptosis induction, autophagy, and cell cycle arrest [14,15]. It has also been shown to augment the anticancer effects of chemotherapy and radiotherapy and mitigate drug resistance [16,17]. In oral squamous cell carcinoma cells, β-elemene has been demonstrated to alleviate cisplatin resistance both in vitro and in vivo by targeting the JAK2/STAT3 pathway [18]. Additionally, β-elemene increases the sensitivity of osteosarcoma cells to doxorubicin by suppressing peroxiredoxin-1 [19]. In gastric cancer, β-elemene impedes the metastasis of multidrug-resistant cells through modulation of the miR-1323/Cbl-b/EGFR pathway [20]. Moreover, β-elemene enhances susceptibility to cisplatin in ovarian carcinoma cells by downregulating ERCC-1 and XIAP and inactivating JNK [21]. Notably, β-elemene also inhibits stemness, promotes differentiation, and disrupts chemoresistance to temozolomide in glioblastoma stem-like cells [22]. These findings collectively highlight the potential of β-elemene as a versatile adjunct to cancer-treatment strategies to address drug resistance in various cancer types.

Long noncoding RNAs (lncRNAs), exceeding 200 bp in length, are implicated in numerous biological functions, including resistance to tamoxifen [23]. The lncRNA H19, an imprinted gene expressed from the maternal allele, is associated with cell proliferation, metastasis, and chemoresistance [24,25]. lncRNA H19 has been identified at elevated levels in gefitinib-resistant cell lines [26]. Our earlier research had demonstrated that curcumenol, the bioactive compound derived from *Curcuma aromatic* Salisb., exerts its ferroptosis-inducing effects in lung cancer cells through the inhibition of lncRNA H19 [27]. Moreover, β-elemene increases the responsiveness of erlotinib in EGFR-mutant NSCLC through the modulation of lncRNA H19-mediated ferroptosis [16]. However, the precise mechanism underlying how lncRNA H19 regulates autophagy and consequently augments the susceptibility of EGFR-mutant cells to gefitinib remains elusive.

Our research illustrates a link between gefitinib resistance and high lncRNA H19 expression, along with abnormal autophagy activation. Our research revealed that β-elemene, when used alongside gefitinib, diminishes cell viability and enhances apoptosis in gefitinib-resistant EGFR-mutant NSCLC cells, specifically HCC827/GR and PC9/GR. The cytotoxic effect of this combination therapy is further enhanced by autophagy inhibitors. This combination therapy lowers lncRNA H19 expression, thereby reversing gefitinib resistance by inhibiting autophagy. Our research emphasizes the potential of β-elemene in enhancing the responsiveness of gefitinib-resistant EGFR-mutant NSCLC cells by regulating autophagy via lncRNA H19. This discovery offers promising avenues for addressing EGFR-TKI therapy resistance in patients with NSCLC.

## 2. Results

### 2.1. Gefitinib Resistance Linked to Dysregulated Autophagy and Elevated lncRNA H19 Levels

**Figure 1:**
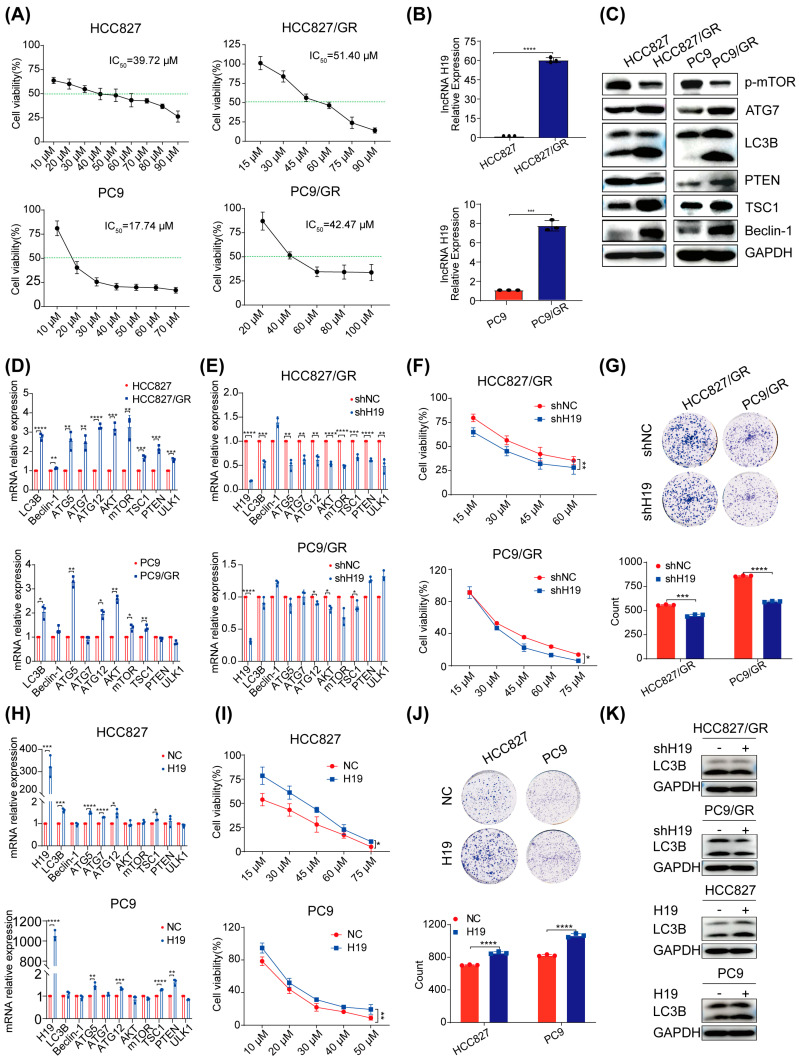
Gefitinib resistance is associated with aberrant autophagy and high expression of lncRNA H19, the green dashed line indicates 50% cell viability. (**A**) CCK8 assay to assess the impact of gefitinib on the viability of sensitive and resistant cell lines. (**B**) qRT-PCR was used to detect the difference of lncRNA H19 in sensitive and resistant cells. (**C**) Western blot analysis to determine the differences in autophagy-related protein expression within sensitive and resistant cells. (**D**) qRT-PCR analysis to examine the autophagy-related gene expression within sensitive and resistant cells. (**E**) Repression of lncRNA H19 expression in resistant cells, followed by qRT-PCR analysis to assess changes in the expression of both lncRNA H19 and autophagy-related genes. (**F**) Repression of lncRNA H19 expression in resistant cells, with subsequent CCK8 assay to evaluate changes in cell sensitivity to gefitinib. (**G**) Repression of lncRNA H19 expression in resistant cells, followed by colony-formation assay to assess cell proliferation capacity. (**H**) Overexpression of lncRNA H19 in sensitive cells, followed by qRT-PCR to detect changes in lncRNA H19 and autophagy-related gene expression. (**I**) Overexpression of lncRNA H19 in sensitive cells, with subsequent CCK8 assay to evaluate changes in cell sensitivity to gefitinib. (**J**) Overexpression of lncRNA H19 in sensitive cells, followed by colony-formation assay to assess cell proliferation capacity. (**K**) Repression of lncRNA H19 expression in resistant cells, or overexpression of lncRNA H19 in sensitive cells, the expression of autophagy protein LC3B was detected via western blot (* *p* < 0.05, ** *p* < 0.01, *** *p* < 0.001, **** *p* < 0.0001, *n* = 3).

By subjecting lung cancer cell lines known for their sensitivity to gefitinib—specifically HCC827 and PC9—to an incremental increase in concentrations of this drug over time (Figure 1A), we were able to generate corresponding resistant variants named as follows: HCC827/GR exhibited a resistance factor of approximately 1.2-fold, while PC9/GR displayed a higher level at around 2.4-fold. Firstly, we found that lncRNA H19 was significantly overexpressed in gefitinib-resistant cells compared with gefitinib-sensitive cells (Figure 1B). Literature reports that lncRNA H19 is closely related to autophagy [28]. Therefore, we detected autophagy-related genes and proteins and found that autophagy was abnormally activated in gefitinib-resistant cells (Figure 1C,D). To further explore the relationship between gefitinib resistance, lncRNA H19 expression, and autophagy, we suppressed the levels of lncRNA H19 in resistant cell lines HCC827/GR and PC9/GR. Subsequent examination using qRT-PCR demonstrates a notable reduction in autophagy activity as evidenced by decreased levels of key autophagy genes (Figure 1E). Moreover, experiments measuring cell viability and colony formation indicate that reducing lncRNA H19 expression not only enhances sensitivity to gefitinib but also impairs proliferation ability in resistant cells (Figure 1F,G). Conversely, increasing the expression of lncRNA H19 in sensitive lung cancer cell lines yields opposite effects (Figure 1H–J). Moreover, knockdown of lncRNA H19 in drug-resistant cells led to a reduction in the expression of LC3B protein, whereas overexpression of lncRNA H19 in sensitive cells resulted in an elevation of LC3B protein levels (Figure 1K). These findings collectively underscore the crucial role played by abnormal autophagy activity along with upregulation of lncRNA H19 in contributing to the development of gefitinib resistance.

### 2.2. Synergistic Effects of β-Elemene and Gefitinib on Apoptosis in Drug-Resistant Cells

**Figure 2:**
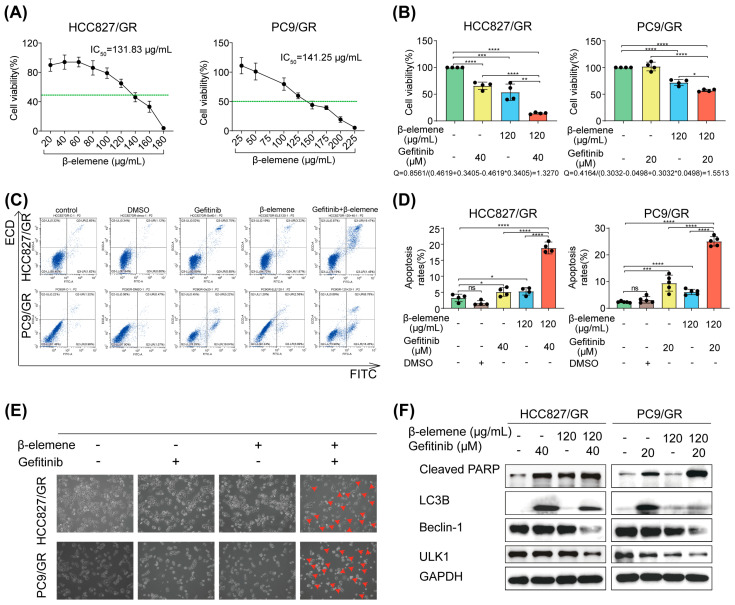
The combination of β-elemene and gefitinib induces apoptosis in drug-resistant cells. (**A**) The viability of resistant cell lines was assessed using a CCK8 assay to evaluate the impact of β-elemene, the green dashed line indicates 50% cell viability. (**B**) A CCK8 assay was conducted to assess the effect of combining β-elemene with gefitinib on the viability of resistant cell lines. (**C**,**D**) Cell apoptosis was detected using flow cytometry analysis. (**E**) Cell morphology examination was performed under a 10× microscope. (**F**) Western blot analysis determined the expression levels of proteins associated with apoptosis and autophagy (* *p* < 0.05, ** *p* < 0.01, *** *p* < 0.001, **** *p* < 0.0001, *n* = 3). HCC827/GR: 120 µg/mL β-elemene and 40 µM gefitinib. PC9/GR: 120 µg/mL β-elemene and 20 µM gefitinib.

To elucidate the influence of β-elemene on overcoming gefitinib resistance, we first examined its impact on the viability of resistant cells at various concentrations using the CCK8 assay. We then explored the synergistic potential of β-elemene and gefitinib by employing Jin’s method, identifying the optimal synergistic concentrations for HCC827/GR cells as 120 µg/mL β-elemene and 40 µM gefitinib, and for PC9/GR cells as 120 µg/mL β-elemene and 20 µM gefitinib, with both combinations yielding a Q-value greater than 1 (Figure 2A,B). Notably, the double treatment significantly increased apoptosis in the drug-resistant cell line, with atrophy and rounding of the cell morphology (Figure 2C–E). The results were further validated through western blot analysis, which demonstrated a notable rise in the apoptosis marker Cleaved PARP after combined treatment. Interestingly, although gefitinib monotherapy elevated the levels of autophagy markers LC3B, Beclin-1, and ULK1 compared to the control group, their expression declined following combined treatment (Figure 2F). In combination, the results highlight the significant collaborative effect of β-elemene and gefitinib in promoting apoptosis in gefitinib-resistant cells.

### 2.3. Synergistic Antitumor Activity of β-Elemene and Gefitinib in Lung Cancer Models In Vivo

**Figure 3:**
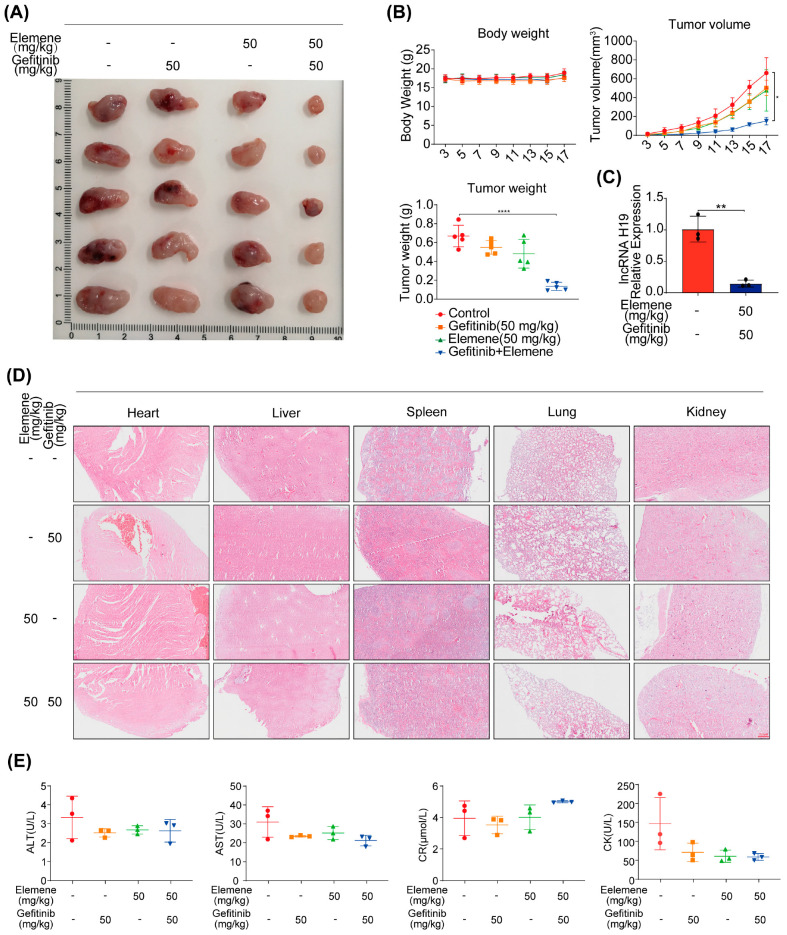
The combination of elemene and gefitinib inhibits tumor cell growth in vivo. A total of 50 mg/kg elemene and 50 mg/kg gefitinib were used alone or in combination. (**A**) Tumor images. (**B**) Statistical graph of nude mouse weights, tumor volume, and tumor weight. (**C**) qRT-PCR analysis was conducted to assess the levels of lncRNA H19 expression in tumor samples. (**D**) HE staining images of heart, liver, spleen, lung, and kidney, the scale is 200 µM. (**E**) Three mice were selected from each group to test serum biochemical indicators (* *p* < 0.05, ** *p* < 0.01, **** *p* < 0.0001, *n* = 3).

To comprehensively evaluate the combined antitumor efficacy of elemene and gefitinib against lung cancer in a living model, we established a xenograft tumor model using HCC827/GR cells. Our results demonstrated that monotherapy with either 50 mg/kg elemene or 50 mg/kg gefitinib had minimal impact on tumor size, whereas the combination treatment significantly suppressed tumor growth (Figure 3A). There were no notable variances in the groups’ body weight, whereas the tumor volume and weight exhibited a significant decrease in the combination treatment group compared to the control group (Figure 3B). qRT-PCR analysis revealed that the combination treatment markedly inhibited lncRNA H19 expression within the tumor (Figure 3C). Furthermore, the utilization of HE staining on vital organs such as the heart, liver, spleen, lung, and kidney did not exhibit any discernible changes in morphology when compared to the control group (Figure 3D). Furthermore, biochemical analyses of serum biomarkers including alanine aminotransferase (ALT), aspartate aminotransferase (AST), serum creatinine (CR), and creatine kinase (CK) indicated no significant differences between groups relative to controls, thus supporting safety considerations associated with this therapeutic regimen (Figure 3E). Collectively, these results underscore the potent synergistic potential of β-elemene and gefitinib in inhibiting lung cancer progression in vivo, offering promising avenues for therapeutic intervention.

### 2.4. β-Elemene Counteracts Gefitinib-Induced Protective Autophagy in Lung Cancer Cells

**Figure 4:**
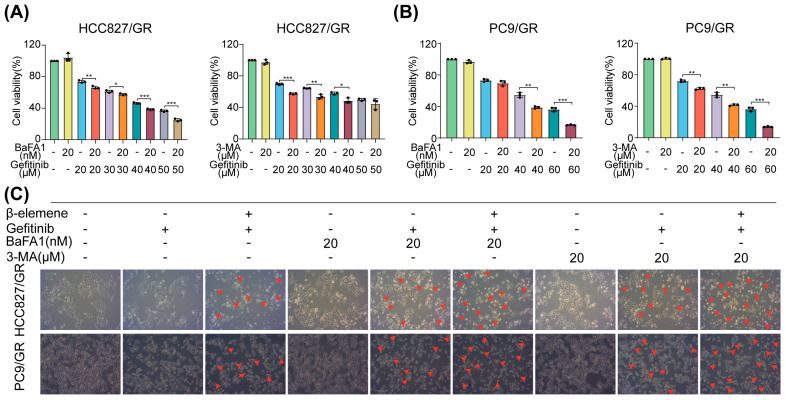

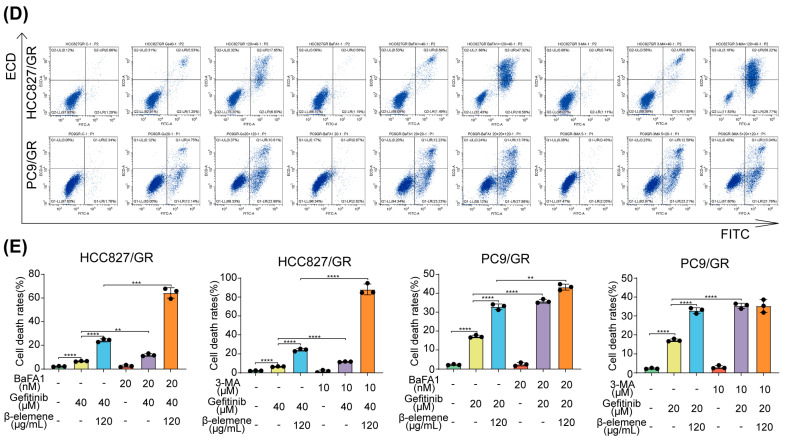
β-elemene can inhibit gefitinib-induced protective autophagy as an autophagy inhibitor and thus exert its anti-lung cancer effect. (**A**,**B**) The CCK8 assay was performed to assess the impact of autophagy inhibitors on the sensitivity of resistant strains to gefitinib, with or without their addition. (**C**) Cell morphology was examined under a 10× microscope. (**D**,**E**) Flow cytometry analysis was performed to assess the impact of autophagy inhibitors on cell demise triggered by β-elemene and gefitinib individually or in conjunction (* *p* < 0.05, ** *p* < 0.01, *** *p* < 0.001, **** *p* < 0.0001, *n* = 3). HCC827/GR: 120 µg/mL β-elemene, 40 µM gefitinib, 20 nM BaFA1, and 10 µM 3-MA. PC9/GR: 120 µg/mL β-elemene, 20 µM gefitinib,20 nM BaFA1, and 10 µM 3-MA.

To further illuminate the intricate mechanisms underlying autophagy triggered by gefitinib in resistant lung cancer cells, we implemented a multi-pronged approach utilizing autophagy blockers Bafilomycin A1 (BaFA1) and 3-Methyladenine (3-MA), individually and in various combinations with gefitinib. This comprehensive strategy was designed to help decipher the precise role of autophagy in the context of gefitinib resistance. Initial experiments using CCK8 assays revealed that both BaFA1 and 3-MA significantly enhanced the cell mortality induced by gefitinib (Figure 4A,B). This finding prompted us to explore the potential of these autophagy inhibitors in conjunction with gefitinib, alone or in combination with β-elemene, in further experiments. In the subsequent experiments, we treated cells with BaFA1 or 3-MA singly, in combination with gefitinib, or in conjunction with both gefitinib and β-elemene. The results demonstrated that autophagy inhibitors, when used in conjunction with gefitinib and β-elemene, were particularly effective in promoting Cell death in resistant cells (Figure 4C–E). This was particularly notable in the group that received a triple combination of autophagy inhibitors, gefitinib, and β-elemene, which exhibited the most robust cell death response.

These observations suggest that gefitinib, in addition to its anticancer effects, may also prompt a protective autophagy mechanism that contributes to cellular resistance. However, β-elemene, when used in combination with autophagy inhibitors, effectively counteracts this mechanism, thereby enhancing the anticancer effects of gefitinib. This mirroring of action between β-elemene and autophagy inhibitors like BaFA1 and 3-MA highlights the potential of using such combinations in future cancer-treatment strategies.

### 2.5. Elucidating β-Elemene’s Modulation of lncRNA H19, Autophagy, and Gefitinib Resistance Dynamics

**Figure 5:**
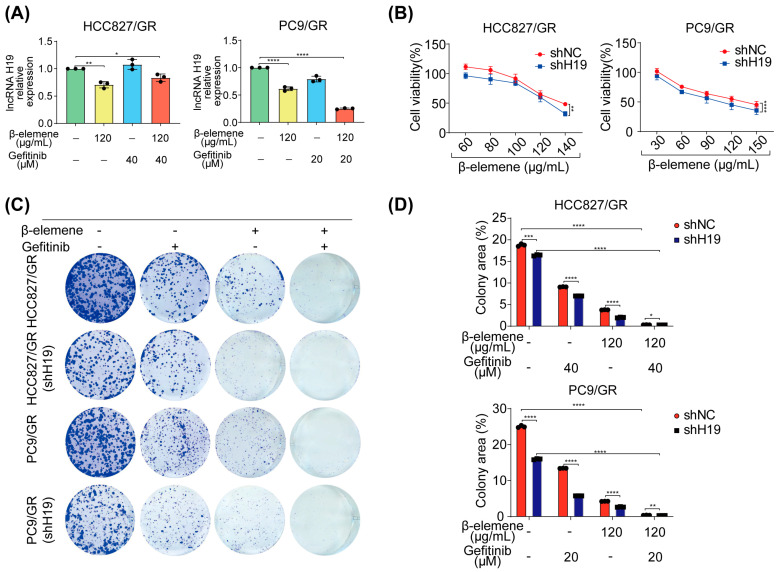

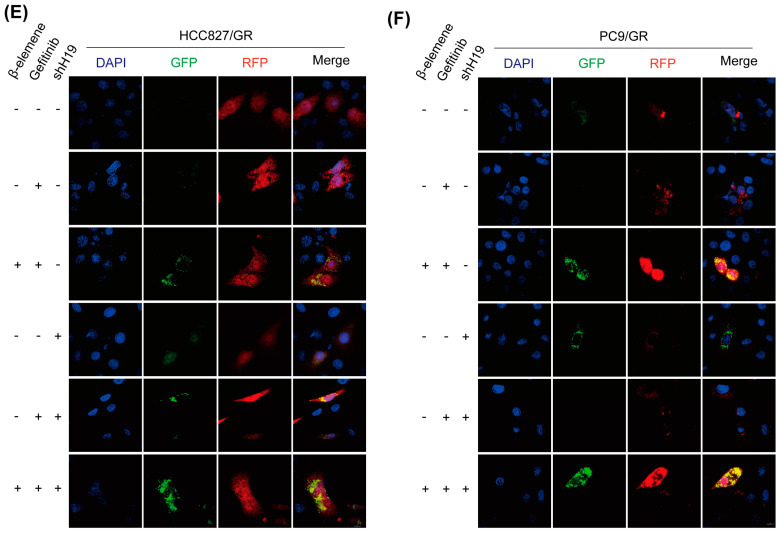
The impact of β-elemene on lncRNA H19, autophagy, and gefitinib resistance. (**A**) qRT-PCR was used to investigate the impact of β-elemene and gefitinib, either alone or in combination, on the expression of lncRNA H19. (**B**) Knockdown of lncRNA H19 was performed, followed by a CCK8 assay to evaluate the sensitivity of resistant cells towards β-elemene. (**C**,**D**) Colony-formation assays were conducted after knockdown of lncRNA H19 to assess the effect of drugs on cell proliferation. (**E**,**F**) The influence of β-elemene and gefitinib alone or in combination on autophagy flux was evaluated following knockdown of lncRNA H19, the scale is 8 µM (* *p* < 0.05, ** *p* < 0.01, *** *p* < 0.001, **** *p* < 0.0001, *n* = 3). HCC827/GR: 120 µg/mL β-elemene and 40 µM gefitinib. PC9/GR: 120 µg/mL β-elemene and 20 µM gefitinib.

The extensive investigation conducted in this study aimed to assess the impact of β-elemene on the expression of lncRNA H19, autophagic mechanisms, and its potential role in overcoming gefitinib resistance in lung cancer cells. We administered β-elemene alone or in combination with gefitinib to HCC827/GR and PC9/GR cells, resulting in a significant decrease in the expression of lncRNA H19 in the resistant cell lines (Figure 5A). To elucidate the functional role of long noncoding RNA (lncRNA) H19 in β-elemene treatment, we employed knockdown strategies to silence lncRNA H19 in drug-resistant strains. Remarkably, our findings demonstrate that silencing lncRNA H19 significantly enhances cellular sensitivity towards β-elemene (Figure 5B). Furthermore, the suppression of lncRNA H19 substantially impedes post-treatment colonization ability of cells, irrespective of single or combination drug administration (Figure 5C,D).

In order to quantitatively evaluate the autophagy flux, we utilized a dual fluorescence reporter gene called GFP-RFP-LC3B. The presence of red fluorescence indicates the existence of autolysosomes, while yellow fluorescence represents autophagosomes. When autophagy flow is unobstructed, the intensity of red fluorescence increases and green fluorescence weakens. Conversely, when there is an impediment in autophagy flow, fusion between autophagosomes and lysosomes is hindered, resulting in an elevation in yellow fluorescence. Our findings demonstrated that treatment with gefitinib alone primarily enhanced the intensity of red fluorescence, indicating an increase in the formation of autolysosomes. However, co-administration of β-elemene and gefitinib led to intensified green fluorescence overlapping with red fluorescence to generate a yellow signal indicative of suppressed activity in the process of autophagy. Furthermore, the co-administration of β-elemene and gefitinib exhibited an augmented suppression of autophagy in cells with depleted lncRNA H19. This is demonstrated by a significant increase in yellow fluorescence intensity and inhibition of autophagosome-lysosome fusion (Figure 5E,F). These insights collectively demonstrate β-elemene’s role in thwarting late-stage autophagy and highlight lncRNA H19 as a pivotal target for reversing gefitinib resistance in lung cancer, with its inhibition potentiating the therapeutic efficacy of β-elemene.

### 2.6. β-Elemene Inhibits EGFR Degradation Mediated by lncRNA H19-Associated Autophagy

Rab7, a small GTP enzyme belonging to the Rab family, is associated with endosomes and lysosomes. Its main role involves facilitating the maturation of endosomes, transportation from late endosomes to lysosomes, and regulating the movement of endosomes and lysosomes along the cytoskeleton for proper localization. Consequently, Rab7 plays a crucial part in controlling various transport processes such as biosynthesis of lysosomal-related organelles (LROs), phagosomes, autophagosomes, and other components related to lysosome formation [29,30]. New findings indicate that the peripheral migration of TKI-sensitive EGFR may be facilitated by Rab7 ubiquitination in the presence of gefitinib, potentially hindering the effectiveness of TKIs. Additionally, gefitinib triggers autophagy-mediated degradation of EGFR in lung cancer cells resistant to TKIs as a protective mechanism, thereby promoting the development of resistance driven by T790M mutation [31]. In our research, we propose that β-elemene, a naturally occurring compound, can potentially counteract this resistance by interfering with the autophagic degradation of EGFR. It does so by reducing the levels of lncRNA H19, which is associated with autophagy. By doing so, β-elemene impedes the degradation of EGFR, thereby preserving its integrity. Furthermore, our research suggests that β-elemene facilitates the relocation of EGFR to the plasma membrane, through the action of Rab7. This relocation is crucial as it helps in countering the resistance developed towards gefitinib, a commonly used TKI.

**Figure 6:**
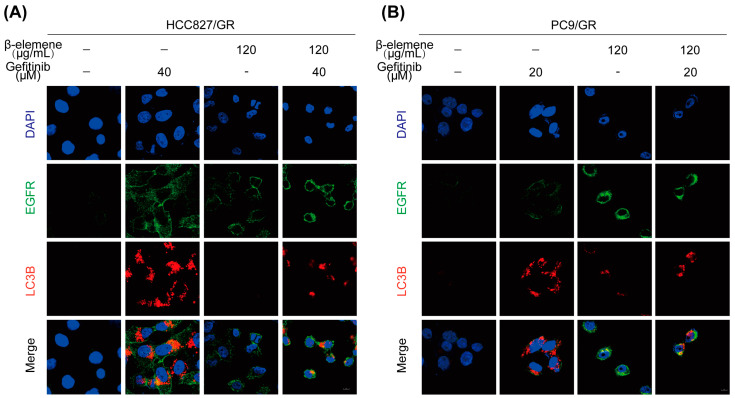

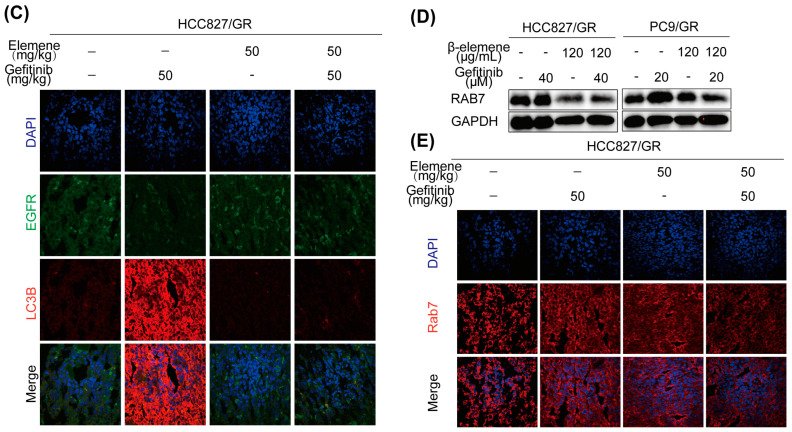
β-elemene inhibits EGFR degradation mediated by lncRNA H19-associated autophagy in both tumor tissues and resistant cells. (**A**,**B**) Immunofluorescence detection of EGFR and LC3B protein expression in HCC827/GR and PC9/GR cells, the scale is 8 µM. (**C**) Immunofluorescence was used to detect EGFR and LC3B protein expression in tumor tissues, and images were captured using a 10× laser confocal microscope. (**D**) Western blot analysis of Rab7 protein expression in HCC827/GR and PC9/GR cells. (**E**) Immunofluorescence was used to detect Rab7 protein expression in tumor tissues, and images were captured using a 10 × laser confocal microscope.

To validate our hypothesis, we performed an immunofluorescence analysis on HCC827/GR and PC9/GR cells, which have been reported to exhibit resistance towards gefitinib. The findings demonstrated a notable elevation in the fluorescence intensity of LC3B protein, a reliable marker for autophagy, upon co-treatment with β-elemene and gefitinib. This increase was mitigated by β-elemene, suggesting that it indeed inhibits autophagy. Furthermore, our results indicate that the distribution of EGFR in the cytoplasm was uniform in the group treated with gefitinib alone. However, when β-elemene was administered, there was an observed translocation of EGFR to the plasma membrane (Figure 6A,B). This finding was further supported by analyzing tumor specimens (Figure 6C), providing additional evidence for our hypothesis. In gefitinib-resistant cells and tumor tissues, we observed an upregulation of Rab7 protein expression in the group treated with gefitinib alone. This increase may contribute to resistance by promoting degradation of EGFR. Interestingly, treatment with β-elemene resulted in a reduction of Rab7 levels and subsequently enhanced sensitivity to gefitinib through relocation of EGFR (Figure 6D,E). Taken together, these findings highlight the potential efficacy of β-elemene as a novel approach to overcome lncRNA H19-mediated autophagy-induced degradation of EGFR and combat resistance to gefitinib in lung cancer.

## 3. Discussion

Globally, the majority of cancer-related deaths are attributed to lung cancer [32], with around 50% of Asian individuals diagnosed with NSCLC showing variations in the EGFR [2,33]. The primary therapeutic approaches primarily rely on gefitinib, a first-generation EGFR TKIs, with the objective of improving patient survival results [34]. Nonetheless, the challenge of inherent and emergent resistance curtails the sustained effectiveness of EGFR TKIs [35]. The most common mechanisms of acquired resistance to EGFR TKIs fall into three categories: target gene modifications, bypass pathway activation, and histological or phenotypic transformation [5]. However, tumor heterogeneity can lead to different oncogenic driver mutations or resistance mechanisms. Therefore, it is important to identify clear tumor resistance mechanisms. In addition, Western medicine lacks effective methods to address EGFR-TKIs resistance. Traditional Chinese medicine (TCM), on the other hand, offers unparalleled advantages in overcoming EGFR-TKIs resistance and preventing secondary or even tertiary resistance. Therefore, the search for TCM that can reverse or delay EGFR-TKIs resistance is an urgent clinical issue that needs to be addressed.

There is growing evidence highlighting the critical roles of autophagy in drug resistance. On one hand, autophagy plays a role in hindering tumorigenesis by removing oncogenic triggers and maintaining genomic stability [36]. On the other hand, once a tumor is established, autophagy becomes a survival mechanism for cancer cells, enabling them to adapt to challenging microenvironments such as hypoxia, metabolic stress, or cell death induced by therapeutic drugs [37]. In this context, autophagy confers cancer cells with drug resistance capabilities and promotes cancer progression. The abnormal activation of autophagy has previously been associated with the emergence of resistance to targeted treatments in cancer, such as gefitinib in NSCLC [38,39]. Consistent with these findings, our results demonstrate that gefitinib treatment alone induces protective autophagy, thereby facilitating cellular resistance. 

In recent years, numerous pharmacological studies have explored the use of autophagy inhibitors as a therapeutic strategy for cancer [40,41]. Most of these studies have demonstrated that inhibiting autophagy can sensitize cancer cells to chemotherapy-induced cell death. This finding is supported by our observation that the co-administration of autophagy inhibitors, including BaFA1 and 3-MA, significantly amplifies apoptosis in resistant cells when used together with gefitinib. Interestingly, β-elemene, when used in conjunction with gefitinib, mirrors the effects of these autophagy inhibitors, suggesting its potential role as an autophagy modulator.

lncRNA H19 is an oncofetal transcript pivotal in the development and progression of various cancers [42,43,44]. It exhibits anti-apoptotic, pro-proliferative, and pro-migratory functions, impacting carcinogenesis from multiple angles. H19 plays a central role in inducing chemoresistance in breast cancer, lung cancer, glioma, liver cancer, and other malignancies [24]. Mechanisms involving lncRNA H19 in chemoresistance include inducing EMT, activating oncogenic signaling pathways, and altering the tumor microenvironment [45,46,47,48]. The literature underscores the complexity of lncRNA H19′s involvement in cancer biology, particularly its association with autophagy and drug resistance mechanisms [49]. Our research findings indicate that gefitinib resistance is not only associated with aberrant autophagy but also correlates with the high expression of lncRNA H19. However, the specific molecular pathways through which lncRNA H19 influences autophagy in the setting of gefitinib resistance are yet to be fully unraveled. Our findings hint at a connection between lncRNA H19 and autophagy in resistant lung cancer cells, pointing towards an intricate regulatory network that awaits further elucidation. 

Moreover, the role of Rab7 in the degradation pathway of EGFR, particularly in the context of β-elemene’s action, introduces additional complexity. Rab7 is implicated in late endosomal trafficking and has been linked to the modulation of EGFR degradation. It has also been reported that PTEN regulates late EGFR endocytosis and degradation by dephosphorylating Rab7 [50]. Although our study suggests a potential interaction between β-elemene, Rab7, and EGFR trafficking that influences gefitinib resistance, conclusive evidence establishing Rab7′s direct involvement in this process is absent, highlighting a critical area for future research.

**Figure 7:**
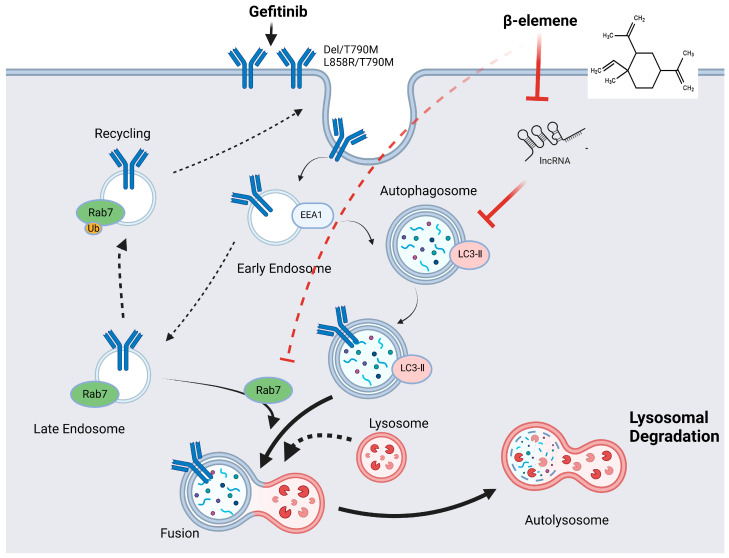
This model elucidates EGFR trafficking, signaling, and autophagy in gefitinib-resistant NSCLC cells following treatment with a combination of β-elemene and gefitinib.

Our research conclusively shows that β-elemene can counteract gefitinib resistance in lung cancer cells by suppressing autophagy linked to lncRNA H19 and halting the autophagy-driven degradation of EGFR. In TKI-resistant lung cancer cells, the administration of gefitinib induces autophagy-mediated degradation of EGFR as a protective mechanism against its targeting, resulting in resistance that is dependent on the T790M mutation. β-elemene inhibits lncRNA H19-mediated autophagy, thereby preventing EGFR degradation and reversing gefitinib resistance (Figure 7). Our study provides valuable findings on the intricate mechanisms by which β-elemene affects the sensitivity of lung cancer cells to gefitinib, highlighting the significant role played by lncRNA H19 and autophagy in this specific context.

## 4. Materials and Methods

### 4.1. Cell Culture

Human NSCLC cell lines PC9 and HCC827 were purchased from ATCC (Manassas, VA, USA) and cultured in our laboratory without any mycoplasma contamination. These cell lines served as the foundation for establishing gefitinib-resistant lines PC9/GR and HCC827/GR, which were developed by subjecting them to escalating concentrations of gefitinib over a period of approximately six months [10]. The cells were grown in a culture medium supplemented with 10% fetal bovine serum and maintained at a temperature of 37 °C in the presence of an atmosphere containing 5% CO_2_.

### 4.2. Reagentd and Antibodies

β-elemene, gefitinib, Bafilomycin A1 (BaFA1), and 3-Methyladenine (3-MA) were acquired from Sigma-Aldrich (#63965, St. Louis, MO, USA) and MedChemExpress (#HY-50895, #HY-16592, #HY-19312, Monmouth Junction, NJ, USA) respectively. The Cell Counting Kit-8 (CCK-8) (#MA0218) was sourced from Meilunbio (Dalian, China), and the Annexin V-FITC Apoptosis Detection Kit (#40302ES60) from YEASEN (Shanghai, China). The PrimeScript RT reagent kit (#Q311) was purchased from Vazyme (Nanjing, China). Antibodies for GAPDH (#5174), LC3B (#83506), ATG7 (#2631), PTEN (#9552), TSC1 (#6935), Beclin-1 (#3738), p-mTOR (#5536), and Rab7 (#95746) were acquired from Cell Signaling Technology (Danvers, MA, USA). GenePharma (Shanghai, China) supplied the plasmids NC, lncRNA H19, lv3-shNC, and lv3-shH19. Lipofectamine™ 2000 was acquired from Thermo Fisher Scientific (Waltham, MA, USA).

### 4.3. Cell Viability Assay

In vitro cellular proliferation was evaluated by means of the CCK-8 assay, adhering to the supplied protocol [27]. Growth curves were plotted using normalized OD490 values. Cell survival rate was quantified by the percentage change in absorbance pre- and post-varied treatment conditions.

### 4.4. Cell Growth Evaluation

The cells were evenly distributed across six-well plates at a density of 1000 cells per well and allowed to incubate for a period of 72 h. Subsequently, they were exposed to β-elemene and/or gefitinib, while refreshing the culture medium every three weeks. After a period of 14 days, the surviving cells were subjected to staining using a solution containing 0.5% crystal violet (60506ES60, YEASEN, Shanghai, China), followed by colony counting utilizing ImageJ software (ImageJ 1.54h version; NIH) [27].

### 4.5. Apoptosis Analysis

Roughly 200,000 cells were gathered and placed in a solution of 100 μL of binding buffer. Subsequently, they underwent simultaneous staining with Annexin-V FITC (3 μL) and PI (5 μL) for a duration of 15 min, maintaining room temperature and avoiding light exposure [27]. Apoptosis was detected using a Beckman Flow Cytometer (Brea, CA, USA).

### 4.6. Western Blot Analysis

Cell extracts were obtained using RIPA buffer (P0013B, Beyotime, Shanghai, China) supplemented with phenylmethylsulfonyl fluoride (#8553, CST). Protein concentrations were determined by means of the BCA Protein Assay Kit (P0009, Beyotime). After electrophoresis, extracts mixed with 5× SDS-PAGE protein sample buffer (P0015, Beyotime) were transferred onto PVDF membranes (Merck Millipore, Billerica, MA, USA). The membranes underwent blocking and incubation with primary and secondary antibodies before being visualized through an ECL system (Bio-Rad, Hercules, CA, USA) [27]. To ensure precision, all procedures were carried out in triplicate.

### 4.7. Quantitative Real-Time RT-PCR 

For the purpose of quantitative real-time RT-PCR, Trizol reagent (15596018, Invitrogen, Thermo Fisher Scientific, Carlsbad, CA, USA) was utilized to extract RNA from cell lines. The NanoDrop 2000c instrument (Thermo Scientific, Waltham, MA, USA) was employed to evaluate the quantity of isolated RNA. To convert RNA into cDNA, the HiScript II 1st Strand cDNA Synthesis Kit (+gDNA wiper) (R212, Vazyme, Nanjing, China) was employed. Quantitative PCR, facilitated by ChamQ SYBR qPCR Master Mix (Q311, Vazyme) and a Bio-Rad Real-Time PCR System (Hercules, CA, USA), was used to evaluate mRNA levels. Primer specifications, supplied by TSINGKE (Beijing, China), are listed in Table 1. GAPDH served as the reference gene for RNA quality control, and relative expression was calculated using the 2^−ΔΔCT^ approach [27].

### 4.8. Measurement of Autophagic Flux

To assess autophagic flux, HCC827/GR and PC9/GR cells were transfected with the GFP-RFP-LC3 plasmid using Lipofectamine 2000 as per the manufacturer’s instructions. Following treatment with β-elemene and gefitinib, fluorescent images were captured using a confocal laser scanning microscope (model #FV3000RS, Olympus, Tokyo, Japan). Autophagic flux was evaluated based on the presence of yellow and red puncta [10]. 

### 4.9. Hematoxylin-Eosin Staining

Hematoxylin and eosin (H&E) staining was performed using the Be-yotime kit according to the instructions. Tissue samples from tumors were preserved in 10% formaldehyde for 24 h, then subjected to a series of preparatory steps including dehydration, clearing, infiltration with wax, embedding in paraffin, and sectioning into 5 μm slices. Hematoxylin was applied to the sections for 5 min, followed by a brief 30 s eosin staining. The sections were then rehydrated and secured with neutral mounting medium. Samples were analyzed by three independent evaluators using an Olympus CX23 microscope (Olympus Corporation, Tokyo, Japan) for microscopic imaging.

### 4.10. Immunofluorescence Assay

The tissue in this study were paraffin tissue sections, and the experimental methods were based on previous studies [51]. Immunofluorescence techniques were applied to evaluate EGFR, LC3B, and Rab7 levels in both cell cultures and tissue specimens. Cultured on glass coverslips within 6-well plates, cells reached a density of 300,000 before fixation with methanol. The paraffin in the tumor samples was eliminated through treatment with xylene and ethanol, followed by rehydration. To retrieve antigens, the sections were heated for 15 min in a sodium citrate solution (pH 6.0), and peroxidase activity was neutralized using hydrogen peroxide in methanol at room temperature. To block non-specific binding, a mixture of Normal Goat Serum and TritonX-100 in PBS was applied for an hour. The primary antibodies were utilized to treat cells and tissue sections, allowing them to bind overnight at a temperature of 4 °C. Following PBS washing, the secondary antibodies were administered for a duration of one hour. The DAPI counterstain was applied to the nuclei, and the samples were stored in a dimly lit room at ambient temperature for a short duration. Dilutions for EGFR, LC3B, and Rab7 primary antibodies were 1:200, while the secondary antibody was diluted to 1:400 in PBS containing 1% bovine serum albumin and 0.3% TritonX-100. A Nikon laser scanning confocal microscope (Nikon C2, Tokyo, Japan) was utilized for the final observation and image acquisition of the stained samples.

### 4.11. Lentivirus and Plasmid Transfection

Transient transfection was performed on HCC827 and PC9 cells using either control plasmids or those encoding lncRNA H19. To achieve stable transfection, lv3-shNC or lv3-shH19 lentiviral constructs were introduced into HCC827/GR and PC9/GR cells. After transfection, the cells underwent treatment with puromycin at a concentration of 2 μg/mL to establish the most suitable dosage for consistent integration [27]. The confirmation of successful transfection was achieved through qPCR analysis.

### 4.12. Tumor Xenograft Model In Vivo

The animal experiments were carried out following the authorized protocols by the Animal Care and Use Committee at Hangzhou Normal University (Approval No. 2023-082). Tumor-bearing nude mice were established by inoculating HCC827/GR cells subcutaneously into BALB/c nude mice’s flank region. After a duration of three days, the mice were randomly assigned to four groups and administered intravenous injections of 200 μL PBS, gefitinib (50 mg/kg/day), β-elemene (50 mg/kg/day), or a combination of β-elemene and gefitinib in a manner that concealed group allocation. Measurements of tumor dimensions and body weight were taken bi-daily following the commencement of treatment. The calculation of tumor volume was based on the following formula: (tumor long diameter × tumor short diameter^2^)/2 [17]. At the end of the study, humane euthanasia was performed on mice and their tumors were collected for further analysis using immunofluorescence and Hematoxylin-eosin techniques.

### 4.13. Statistical Analysis

Relevant experimental results were reported as the average ± standard deviation (SD), obtained from three separate trials. Group comparisons were performed using GraphPad Prism version 9.0, employing Student’s *t*-tests for both paired and unpaired analyses. Statistical significance was determined at a *p* value less than 0.05.

## 5. Conclusions

In summary, our study demonstrates that β-elemene effectively overcomes lung cancer resistance to gefitinib by targeting lncRNA H19 and autophagy, thereby enhancing the efficacy of gefitinib. This highlights the role of β-elemene in preventing EGFR degradation and promoting its relocalization to the cell membrane, thereby restoring cellular sensitivity to gefitinib. These findings suggest that β-elemene could serve as a valuable adjunctive therapy in lung cancer-treatment strategies, particularly for overcoming resistance to EGFR-TKIs. However, it is important to acknowledge several limitations in our research. Firstly, the specific molecular mechanisms underlying the interactions among β-elemene, lncRNA H19, and autophagy require further elucidation. Additionally, there is insufficient evidence to confirm Rab7′s direct involvement in the process of β-elemene-mediated reversal of resistance, which may be an important area for future research. Our study provides a theoretical foundation for overcoming EGFR-TKI resistance and developing targeted personalized therapies, which is crucial for translating laboratory research findings into clinical applications.

## Figures and Tables

**Table 1:** Primer Sequence Listing.

Gene	Forward Primer	Reverse Primer
*GAPDH*	5′-GCACCGTCAAGGCTGAGAAC-3′	5′-TGGTGAAGAACGCCAGTGGA-3′
*LncRNA H19*	5′-TCCCAGAACCCACAACATGAA-3′	5′-TTCACCTTCCAGAGCCGATTC-3′
*LC3B*	5′-GAGAAGCAGCTTCCTGTTCTGG-3′	5′-GTGTCCGTTCACCAACAGGAAG-3′
*Beclin-1*	5′-CTGGACACTCAGCTCAACGTCA-3′	CTCTAGTGCCAGCTCCTTTAGC-3′
*ATG5*	5′-GCAGATGGACAGTTGCACACAC-3′	5′-GAGGTGTTTCCAACATTGGCTCA-3′
*ATG7*	5′-CGTTGCCCACAGCATCATCTTC-3′	5′-CACTGAGGTTCACCATCCTTGG-3′
*ATG12*	5′-GGGAAGGACTTACGGATGTCTC-3′	5′-AGGAGTGTCTCCCACAGCCTTT-3′
*AKT*	5′-TGGACTACCTGCACTCGGAGAA-3′	5′-GTGCCGCAAAAGGTCTTCATGG-3′
*mTOR*	5′-AGCATCGGATGCTTAGGAGTGG-3′	5′-CAGCCAGTCATCTTTGGAGACC-3′
*TSC1*	5′-CTGGACAGACTGATACAGCAGG-3′	5′-TGCGGATCTCATCTGAAGGAGG-3′
*PTEN*	5′-TGAGTTCCCTCAGCCGTTACCT-3′	5′-GAGGTTTCCTCTGGTCCTGGTA-3′
*ULK1*	5′-GCAAGGACTCTTCCTGTGACAC-3′	5′-CCACTGCACATCAGGCTGTCTG-3′

## Data Availability

Data contained within the article.
